# Pancreatic islet cell therapy for type I diabetes: understanding the effects of glucose stimulation on islets in order to produce better islets for transplantation

**DOI:** 10.1186/1479-5876-5-1

**Published:** 2007-01-03

**Authors:** Jiaqiang Ren, Ping Jin, Ena Wang, Eric Liu, David M Harlan, Xin Li, David F Stroncek

**Affiliations:** 1 Department of Transfusion Medicine, Clinical Center, National Institutes of Health, Bethesda, MD, 20892, USA; 2 National Institute of Diabetes, Digestive and Kidney Disease, National Institutes of Health, Bethesda, MD, 20892, USA

## Abstract

While insulin replacement remains the cornerstone treatment for type I diabetes mellitus (T1DM), the transplantation of pancreatic islets of Langerhans has the potential to become an important alternative. And yet, islet transplant therapy is limited by several factors, including far too few donor pancreases. Attempts to expand mature islets or to produce islets from stem cells are far from clinical application. The production and expansion of the insulin-producing cells within the islet (so called β cells), or even creating cells that secrete insulin under appropriate physiological control, has proven difficult. The difficulty is explained, in part, because insulin synthesis and release is complex, unique, and not entirely characterized. Understanding β-cell function at the molecular level will likely facilitate the development of techniques to manufacture β-cells from stem cells. We will review islet transplantation, as well as the mechanisms underlying insulin transcription, translation and glucose stimulated insulin release.

## Background

Insulin, the body's only blood glucose-lowering hormone, is exclusively produced by the β-cells of the pancreatic islets of Langerhans. Type I diabetes mellitus (T1DM), formerly known as insulin-dependent diabetes or juvenile diabetes, affects an estimated 1,000,000 Americans, and is thought to result from the destruction of β cells by autologous cytotoxic T cells. The discovery of insulin in the 1920's was a major advance in diabetes treatment – freeing patients from the then "state of the art" starvation diets that, at best, prolonged survival for a few weeks, months, or in very rare cases, a few years. Insulin therapy changed diabetes from a rapidly fatal disease to a chronic disease associated with significant secondary complications, such as renal failure, neuropathy, cardiovascular disease, and retinopathy. While aggressive insulin therapy that maintains glucose levels near the normal range reduces the risk of secondary complications, patients often find such control difficult to achieve and suffer an increased risk of hypoglycemia [1]. Nonetheless, T1DM treatment has significantly improved with affordable glucose monitoring instruments, new insulin formulations, and improved insulin delivery systems.

### Transplantation

Many clinical investigators have attempted β-cell replacement therapy, either in the form of whole pancreas or islet transplantation. Whole pancreas transplantation was first performed in the 1960s, but was a relatively uncommon procedure until the 1980's. From December 16, 1966 to December 31, 2004, more than 23,000 pancreas transplants were reported to the International Pancreas Transplant Registry (IPTR), including > 17000 from the US and almost 6000 from outside the US. The annual number of US in 2004 was close to 1500 [2]. The procedure's increasing popularity can be ascribed to improvements in organ preservation, surgical technique, and immunosuppressive therapy.

Patients given a pancreas transplant, at experienced centers, currently can anticipate a greater than 85% likelihood that they will enjoy insulin independent euglycemia 1 year later, and about 50% will maintain that excellent metabolic outcome 5 years following the transplant. While most pancreas transplant recipients no longer require exogenous insulin for blood glucose control, the procedure has not been shown to decrease the severity or frequency of the secondary complications associated with diabetes. For instance, while pancreas transplant has been shown to reverse histologically defined diabetic nephropathy ten years after transplantation, only a minority of grafts survive that long, and the immunosuppression given to preserve the transplanted organ may be more nephrotoxic than the diabetes prompting the transplant [3]. In fact, whether pancreas transplant alone offers any survival advantage versus insulin therapy alone is debated, and transplant recipient survival may even be worse [4,5].

While pancreas transplantation has yielded promising results, other investigators have worked to develop isolated islet transplantation. Islet transplantation is potentially appealing because the transplant technique does not require major surgery. Using animal models in the 1970s, islets were first isolated from the pancreas and effectively transplanted [6]. Preliminary human islet cell transplant reports appeared in the late 1970's [7,8]. Islet transplantation was not used clinically to treat type I diabetes until 1989 [9].

Although successful procedure of living-donor transplantation of islets has been reported [10], in its current status, most islet transplants involve the isolation of islets from cadaveric (deceased) donor organs. The isolation process entails both enzymatic (using collagenase) and mechanical disruption of a cadaveric pancreas into small fragments. Islets are then purified from the remaining exocrine tissue by density gradient separation [11]. Once "purified" (and isolated islets should more accurately be termed "enriched" because exocrine pancreatic fragments are also nearly always present), the islets are infused into the recipient's portal vein where they lodge in the liver's portal vein tree. Islets have been introduced into the portal circulation through the umbilical vein, but percutaneous transhepatic angiographic infusion is now used most frequently. Isolated islets are usually infused within 48 hours (some centers infuse them within hours), and most recipients require islets from more than one cadaver. The islets isolated from one cadaver are typically given with each infusion, such that most islet recipients end-up requiring two or more islet infusions. While islet infusions are generally well tolerated, they can be complicated by (at least temporary) portal hypertension, thrombosis or hemorrhage.

Before 2000, the preceding 2 decades of islet transplantation by investigators world-wide, had achieved 1-year insulin independence rates of less than 12% [12]. However, in 2000 the group from the University of Alberta, Edmonton reported that their protocol for islet transplantation and immunosuppression significantly improved graft function [13]. The Edmonton protocol used a steroid-free immunosuppressive regimen and high quality islet cells from 2 or more donors. The quality of islet cells obtained from each cadaver was improved by limiting cold ischemia time to 8 hours prior to initiating the isolation procedure and using a standardized collagenase preparation. Further, the Edmonton group transplanted the isolated islets, as soon as possible following the isolation, via transhepatic angiography. To prevent islet allograft rejection, the Edmonton team avoided glucocorticoids by giving the recipient induction immunosuppression with the anti- IL-2 receptor antibody (daclizumab), then more chronic immunosuppression was initiated with sirolimus (rapamycin) and tacrolimus (FK-506) [14,15]. Recently, an international, multicenter trial tested whether islet transplantation using the Edmonton protocol could be generalized. From more than 2000 with T1DM expressing an interest in the protocol, 36 patients were selected and given allogeneic islets. Sixteen of the 36 (44%) attained the primary end point of insulin independence (defined as a fasting blood glucose not to exceed 140 mg/dl more than 3x/week, and a 2 hour post-prandial blood glucose not to exceed 180 mg/dl more than 4x/week), 28% had partial graft function and 28% had complete graft loss. While many of the insulin independent subjects did not have normal blood glucose values by currently accepted criteria, the subjects who reached the primary end point had no severe hypoglycemia or severe hyperglycemia, and those with partial function had a marked benefit in glycemia control in contrast to their baseline status [16]. Unfortunately, and for reasons not well understood, only 14% of the subjects remained insulin independent 2 years after receiving an islet transplant. Further, the current immunosuppressive regimen is associated with heightened risk of infection, β cell toxicity, and (most importantly) nephropathy such that the protocol participants lost, on average about 5% of their kidney function each year.

### Alternative sources of β Islets

While pancreas and islet cell transplants have the potential to treat many patients with diabetes, these transplants require the collection of pancreata from organ donors [13,17], and donors are very limited. Optimistic estimates suggest that in a typical year and using current techniques, islets isolated form U.S. cadaveric pancreata could transplant at most 1,000 to 2,000 patients [14]. In order to treat more patients, the field needs an alternative source of cells capable of physiologically regulated insulin secretion. Transplanting cells generated from stem cells is one potential treatment alternative [18] and another is the expansion adult islet β cells, xenogeneic islet is considered another alternative source.

While a stem cell that could be isolated from the pancreas, expanded, and differentiated *in vitro *into mature beta-like cells may exist, such a cell has not yet been identified [19], and culture techniques have not been perfected. If such a cell could be isolated from the pancreas and expanded *in vitro*, it is possible that enough islets could be produced from a pancreas from a single donor for one or more successful transplants.

Another alternative is to produce β cells from stem cells. Several groups have attempted to differentiate embryonic stem cells into β cells, but none has yet been successful. Islet-like cells have been generated from embryonic stem cells, but the cells do not secrete insulin in a physiologically regulated fashion [20]. In vitro differentiation of embryonic stem cells is to mimic normal embryonic development, the exposure of stem cells to growth factors, extracellular matrix components, and cell-cell interactions may promote and streamline the differentiation process. The existing protocols for generating insulin-producing cells from embryonic stem cells can be divided into spontaneous differentiation and induced differentiation. The efficiency of the former protocol is too low to be of practical value [21-23] and induced differentiation is the mainstream. Lumelsky modified a protocol used to generate neurons from mouse embryonic stem cells and obtained insulin-producing cells [24], their results were repeated by other groups [25,26]. However, some reports showed that the cells can not produce insulin themselves, but rather absorb insulin from the culture medium while undergoing apoptosis [27]. Recently, D'Amour developed a five-stage protocol to differentiate human embryonic stem cells to endocrine hormone-expressing cells through a series of endodermal intermediates resembling those that occur during pancreatic development in vivo [28]. Despite the facts, the procedure of pancreas development is so complex and precise that many aspects are not well understood now. Fortunately, advances in high-throughout technology such as microarray technology will facilitate the discovery of regulating network of pancreatic development, which will provide more information to better direct differentiating stem cells.

Adult stem cells are another potential source of islets [18]. Some animal hematopoietic transplant models have suggested that adult pluripotential stem cells from bone marrow can be induced to transdifferentiate into β-like cells, but subsequent studies have suggested that the cells were the result of cell-to-cell fusion rather than transdifferentiation [29]. It may also be possible to generate insulin producing cells using gene therapy. Some studies have suggested that transfecting hepatocytes with the gene encoding *PDX-1 *resulted in an insulin producing cell, but those results have not been widely reproduced [30-32].

Xenogeneic islets is another alternative; a couple of papers were recently published in Nature Medicine showing that porcine islets xenografts can succeed and has potential for clinical application [33,34]. However, xenogeneic transplants take major immunosuppression which significantly outweighs the risk of diabetes and the results were not consistent, several of the grafts failed early.

While all of these potential therapies are promising, none is yet useful clinically. Furthermore, since β cell insulin release to control glucose levels is both very complex and precise, it is likely that any cultured, expanded, or otherwise manufactured cells will have to function with β cell-like precision. We suggest therefore that it will not be possible to create highly effective expanded, cultured or manufactured β cells until the mechanisms responsible for glucose-induced β cell insulin synthesis and release are completely understood and these functions can be duplicated.

## Insulin transcription, translation, and release

The production of insulin and its release from islets is complex and tightly regulated. Glucose affects insulin at all levels, including transcription, translation and release.

### Transcription

Insulin is encoded by the insulin gene located on chromosome 11p15.5 [35]. Insulin expression is restricted to the β cell and insulin transcription is controlled by the insulin promoter, and in particular a highly conserved 340 bp region located immediately upstream from the transcription initiation start site [36]. The insulin promoter is responsible for tissue specific and metabolic regulation of the insulin gene. The most critical transcription activation elements of the insulin promoter are the A3, C1 and E1 sites.

The most important insulin transcription factor is the homeodomain transcription factor pancreatic/duodenal homebox-1 (PDX-1). In the adult islet, PDX-1 is only expressed in the pancreatic β cells and plays an important role in glucose-stimulated insulin gene transcription. PDX-1 is also essential for maintenance of the β cell phenotype and pancreatic development [36,37]. PDX-1 binds to the A3 box of the insulin promoter. Alone PDX-1 has little activity, but it becomes a potent factor when it interacts with the heterodimer of basic helix-loop-helix (bHLH) proteins which bind to the E1 box. These bHLH heterodimers are made up of a Class A bHLH protein which is expressed ubiquitously in many different tissues and a Class B bHLH protein that is specifically expressed in β cells. The members of the class A bHLH family in the heterodimer that binds to the E1 site are products of the *E2A *gene; E12, E47, and E2/5 and the class B bHLH protein BETA2/NeuroD. The heterodimer E47/BETA2 is found in β cells.

Another important insulin transcription factor is MafA which belongs to the Maf family of transcription factors. This family of transcription factors contains a basic motif followed by a leucine zipper. In addition, MafA also contains an acidic domain that acts as a transcription initiation domain [38]. MafA binds to the C1 site of the insulin promoter. MafA is a glucose-regulated and pancreatic β cell-specific transcriptional activator for the insulin gene [39-41], which produces synergistic activation with PDX-1 or BETA2.

**Figure 1:**
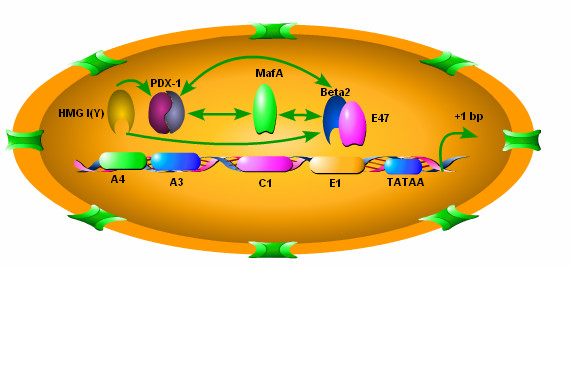
**The promoter region of insulin gene**. The organization of the proximal portion (-340 bp to +1 bp) of the insulin promoter including critical transcription activation elements and their binding transcription factors are shown. The critical transcription activation elements are illustrated as boxes. Above the boxes are shown the names of transcription factors that can bind to corresponding elements. Elements A3, C1, E1 have been implicated in β cell-specific expression of the insulin gene, which is mediated by the restricted cellular distribution of their transcription factors PDX-1, MafA, Beta-2. The synergistic activities of PDX-1, MafA and Beta-2 are illustrated by green arrows. In addition to transcription factors, DNA-binding proteins such as HMG I (Y) can promote the binding of PDX-1 and Beta2/E47 to their corresponding elements.

In addition to PDX1, E47/BETA2/NeuroD, and MafA, other factors influence insulin transcription. Histones and DNA-binding proteins of the high mobility group (HMG) increases the binding of *PDX-1 *and bHLH heterodimers to the A and E sites. One important HMG protein is HMG 1(Y) which binds to the A3/A4 site of the insulin promoter [37]. Other proteins that contribute to insulin transcription include members of the hepatic nuclear factors and PAX families [36] (Figure 1).

### Insulin synthesis

Translating the insulin mRNA leads to preproinsulin production. Approximately 30 to 60 seconds after preproinsulin is synthesized in the ER, the pre portion is removed enzymatically and proinsulin is transported along the microtubule network system in transport vesicles to the cis part of the Golgi apparatus. The proinsulin was first packaged into clathrin-coated immature granules, where proinsulin is further converted to insulin and C-peptide [42]. The clathrin-coated granules then become mature granules, where the insulin crystals are formed; thus the insulin is stored in the mature secretory granules until it is either released by exocytosis or degraded by crinophagy [43].

The β cell has highly developed ER specialized for the synthesis of insulin. β Cells can control the rate of insulin production by regulating insulin synthesis in the ER in response to glucose stimulation. Eukaryotic initiation factor 2 (eIF2) is an important factor regulating protein biosynthesis. The eIF2 factor participates in the formation of translational ternary complex (eIF2-GTP·Met-tRNAi), recruiting charged initiator methionyl-tRNA to the 40S ribosomal subunit. The activity of the eIF2 complex is dependent on its state of phosphorylation. Pancreatic ER kinase (PERK), an important regulator of insulin translation in β cells, phosphorylates eIF2 complex and hence lowers insulin translation [44]. PERK activity is sensitive to glucose levels [45]. Therefore, PERK signaling is particularly important to normal β cell function.

The initiation complex eIF4F initiates the recruitment of the 40S ribosome to mRNA in insulin synthesis. Hypophosphorylated eIF4E-binding proteins (4E-BPs) inhibit eIF4F complex formation, but phosphorylation of 4E-BP1 leads to loss of this inhibition, therefore inducing an increase in mRNA translation [46,47].

### Insulin release

Nearly all insulin released by β cells is from insulin secretory granules. To release insulin, the granules must be recruited from the cytoplasm, translocated to the plasma membrane where they are docked, fuse with the plasma membrane, and release their contents into the extracellular space. A group of proteins known as the SNAp REceptors (SNARES) are important for directing the insulin vesicles to the plasma membrane. The actual docking of the vesicle with the plasma membrane involves the linking of the plasma membrane proteins syntaxin and synaptosomal-associated protein 25 (SNAP-25) with the vesicle protein vesicle-associated protein 2 (VAMP-2) or synaptobrevin-2. Syntaxin and SNAP-25 are known as *t*-SNARE and VAMP-2 is known as v-SNARE.

**Figure 2:**
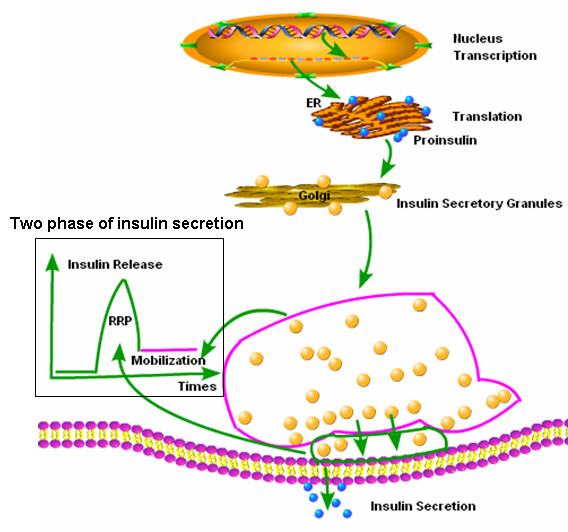
**Insulin synthesis and secretion process**. After preproinsulin mRNA transcription, preproinsulin is synthesized in the ER and converted into proinsulin, proinsulin is transported through the Golgi apparatus and packaged into immature clathrin-coated granules, where proinsulin is processed into insulin and c-peptide. The immature granules can then become mature granules containing cystalized insulin. After glucose stimulation insulin granules exhibit two characteristic phases that consist of a rapidly initiated but transient first phase and a sustained second phase, because the granules are divided into two different pools. (1) A limited pool of granules (< 5%) ready for immediate release and is referred to as the "readily releasable pool" (RRP), which account for the first release phase. (2) Most of the granules (> 95%) belong to a reserve pool responsible for the second-phase of insulin secretion, granules in this pool must undergo mobilization before they can gain release competence.

Insulin granule secretion in response to glucose stimulation exhibits two characteristic phases. This biphasic pattern consists of a rapidly initiated, but transient first phase of insulin release, and a sustained second phase [48-50]. β Cells contain two pools of insulin containing secretory granules that have distinct release processes. A limited pool of granules (< 5%) is available for immediate release and is referred to as the "readily releasable pool" (RRP). However, most of the insulin granules (> 95%) belong to a reserve pool and must undergo mobilization before they can gain release competence [51-53]. The release of RRP granules accounts for the first phase of insulin secretion. The end of the first phase marked the depletion of this pool. The subsequent granule re-supply or mobilization from a reserve pool of granules and release of these mobilized granules is responsible for the second-phase of insulin secretion [54] (Figure 2).

## Effects of glucose on insulin transcription, translation and release

Glucose controls all aspects of insulin regulation. Glucose is the major physiologic regulator of insulin transcription, translational regulation of insulin biosynthesis, and insulin secretion. The response of β cells to glucose is complex. Glucose has both immediate and long term effects that are mediated on several levels including granule release, protein translation and mRNA transcription. β Cells have large stores of insulin in granules and immediately following glucose stimulation a small proportion of these granules are released. To renew these stores, insulin biosynthesis starts immediately and during this period after glucose stimulation glucose-induced insulin biosynthesis is regulated mainly at the translational level [55,56]. During this period glucose metabolism may be coupled to the transcriptional activation of immediate-early response factors. During periods of prolonged glucose stimulation (> 12 h), glucose increases insulin biosynthesis by accelerating insulin gene transcription, as well as by increasing preproinsulin mRNA stability and protein translation.

### Glucose control of insulin secretion

The mechanism by which glucose triggers insulin secretion has been well established. β-cells do not release insulin in response to glucose itself, but to glucose metabolism [57]. Glucose enters β-cells via the GLUT 2 transporter. Intracellular glucose is then metabolized to generate ATP. This results in an increase in the cytosolic ATP: ADP ratio which in turn results in the closure of K_ATP_-channels, membrane depolarization, and initiation of electrical activity [58].

Intracellular Ca^2+ ^signaling plays a critical role in the regulation glucose-mediated insulin secretion. The depolarization of the plasma membrane by the closure of the K_ATP _channels allows the opening of the L-type voltage dependent Ca^2+^-channels (L-VDCC) which results in an increase in the influx of calcium and an increase in the intracellular Ca^2+ ^concentration, an event that causes exocytosis of the insulin granules [52]. This ATP-sensitive K^+ ^(K_ATP_) insulin release pathway is also known as the triggering pathway.

SNARE proteins play a critical role in insulin granule secretion [59,60]. The granule protein, v-SNARE, and the β cell plasma membrane protein, t-SNARE, bring insulin granules in close contact with the plasma membrane and plasma membrane Ca ^2+^-channels [61]. When glucose stimulation leads to the closure of K_ATP _channels and the opening of the Ca ^2+^-channels and the RRP granules located just beneath the inner mouth of Ca ^2+^-channels are exposed to high levels of Ca^2+ ^causing RPR granule release or exocytosis. The release of insulin RPR granules proceeds in an essentially all-or-none fashion depending on whether the Ca ^2+^-channels are open or not [53,54,62,63].

Glucose also affects calcium influx via cyclin-dependent kinase 5 kinase (Cdk5). Cdk5 phosphorylates the α_1C _subunit of L-VDCC which decreases its activity by preventing the binding of L-VDCC to the SNARE proteins. The activity of CdK5 kinase is dependent on glucose. High glucose concentrations inhibit the Cdk5 kinase activity which in turn increases the inward whole-cell Ca^2+ ^channel current and increases Ca^2+^influx, leading to enhanced insulin secretion [64].

Glucose not only triggers insulin secretion but also amplifies it. The amplifying effect of glucose on insulin release is known as the augmentative pathway. This pathway does not cause insulin secretion by itself, but it enhances Ca^2+ ^mediated secretion. The augmentation pathway is independent of K_ATP_-channel inhibition. In fact, changes in ADP concentration mediate the amplifying effect while ATP represents a permissive factor [65,66].

**Figure 3:**
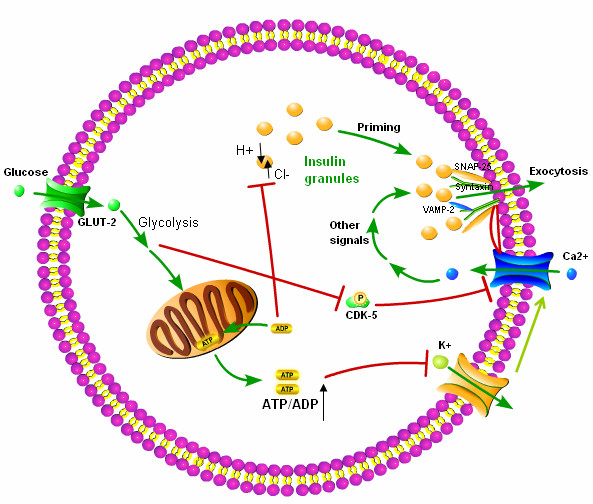
**Glucose control of insulin secretion**. Glucose-stimulates insulin secretion via two mechanisms: the triggering pathway and the amplifying pathway. (1) Glucose enters beta cells through GLUT-2 and undergoes glycolysis. This metabolism increases the ratio of ATP to ADP, which inhibits the ATP-sensitive K_ATP_-channels, leading to membrane depolarization and opening of voltage-dependent calcium channels (VDCC), with a resultant major increase in cytosolic calcium, which, in turn, triggers exocytosis. SNARE proteins play a critical role in insulin granule secretion. The linking of the plasma membrane proteins syntaxin and SNAP-25 to vesicle protein VAMP-2/synaptobrevin-2 cause the docking of the vesicle, bringing insulin granules in close contact with the plasma membrane and calcium channels, after opening of calcium channels, the readily releasable pool (RRP) insulin granules located nearby are exposed to high level of Ca2+, resulting in RRP granule exocytosis. CDK5 can inhibit VDCC activity by phosphorylating its subunit, thus inhibiting insulin secretion; however, high glucose concentrations inhibit the Cdk5 kinase activity which in turn increases Ca^2+ ^influx, leading to enhanced insulin secretion. (2) Insulin granules in reserve pool must undergo acidification to gain secretion competence. This mobilization or priming process is dependent on the simultaneous operation of a V-type H^+^-ATPase and ClC-3 Cl^- ^channels. Cl^- ^uptake determines the extent of granular acidification by providing a counter-ion required to allow continuous H^+ ^pumping. ADP can inhibit Cl- channel activity, however, glucose metabolism reduces the ADP level, leading to the loss of inhibition to Cl- channels, so insulin secretory granules undergo acidification and the secretion process is augmented.

Before insulin secretory granules can be released, they must be primed or acidified. Insulin granule priming is dependent on the simultaneous operation of a V-type H^+^-ATPase and ClC-3 Cl^- ^channels. Cl^- ^ion uptake determines the extent of granular acidification by providing a counter-ion required to allow continuous H^+ ^pumping. The activity of the ClC-3 Cl^- ^channels is inhibited by high concentration of ADP. As a result of glucose metabolism, the ADP level is decreased while ATP level is increased, leading to granular acidification. Acidification is a prerequisite for insulin secretion and only after acidification, can granules undergo exocytosis whenever Ca ^2+ ^influx increases to exocytotic levels and thus insulin secretion is amplified [67,68] (Figure 3).

### Glucose control of insulin transcription

The β-cells contain a large pool of cytoplasmic insulin mRNA which makes up 10 to15% of all the β cells' total mRNA [69]. Even at low glucose concentrations large quantities of insulin mRNA are present, however, at low plasma glucose concentrations (< 3 mM) the insulin mRNA reservoirs are due to basal insulin gene transcription. At higher glucose concentrations insulin transcription increases. For example, the mouse insulin gene promoter is transcriptionally active in the absence of glucose, but the addition of glucose stimulates transcription about 3-fold after 10 minutes, and the stimulatory effect is most pronounced 30 minutes after the glucose stimulus, but declines thereafter [70]. However, the regulation of insulin mRNA production is complex. No one factor completely controls insulin gene expression and transcription continues even when components involved in the stimulus-dependent up-regulation of insulin gene transcription are blocked or even knocked-out.

In addition to increasing the rate of insulin mRNA transcription, glucose also prolongs the half-life of preproinsulin mRNA. The half-life of preproinsulin mRNA in cells incubated in 17 mM of glucose is 76.8 hours, however, the half-life of those islets incubated in 3.3 mM is 29.0 hours [71]. Other studies have found that in rat islets lowering glucose from 11 mM to 2 mM decreased insulin mRNA levels by 38%, 79% and 66% at 3, 6 and 12 hours respectively [72]. Another study found that the content of preproinsulin mRNA in islets cultured at 3.3 mM glucose was reduced to 10% of the control islets after 24 hours and remained at that level for up to 7 days of incubation [73]. This suggests that the lower glucose concentrations may increase the proinsulin mRNA degradation rate.

The proinsulin mRNA 5' and 3' untranslated regions (UTR) also act cooperatively to increase glucose-induced preproinsulin biosynthesis [74]. Elements within the 3' UTR stabilize the mRNA and those within the 5' UTR stimulate preproinsulin translation. In addition, insulin gene 5'-untranslated region alternative splicing has been found in isolated human pancreatic islets, and the alternatively sliced mRNA is translated twice as efficiently (*in vitro*) as the native proinsulin mRNA. Twenty-four hours of hyperglycemia increases the expression of the alternatively spliced form by more than 2-fold and 72 hours of hyperglycemia increases it by more than 10-fold, suggesting that prolonged exposure to high glucose concentrations may indirectly affect the insulin splicing process and lead to the preferential use of the cryptic splice site [75].

The effect of short-term glucose stimulation on preproinsulin transcription is controversial. Isolated rat islets exposed to 16.7 mM glucose for 1 hour and 3 hours did not alter proinsulin mRNA levels, however, longer incubations (6, 12, and 24 hours)increased proinsulin mRNA levels 1.7-, 2.2-, and 2.6-foldi [76]. Similarly, incubating rat islets for 1 hour in 16.7 mM glucose, compared to 2.8 mM glucose, resulted in similar preproinsulin mRNA levels [77]. However, another study of rat islets found that proinsulin biosynthetic rates increased within 1 hour of exposure to high glucose concentrations, with fifty percent of this increase attributed to preproinsulin gene transcription. Furthermore, these investigators found that the glucose stimulatory effect was short-lived; the transcriptional activity was maximal at 30 minutes but markedly decreased thereafter [78].

The apparently contradictory results may be due to the dynamics of newly synthesized proinsulin mRNA, which undergoes faster degradation than pre-existing proinsulin mRNA [78]. Perhaps during 1-hour glucose stimulation, newly synthesized preproinsulin mRNA is degraded so rapidly that changes in preproinsulin mRNA levels are not detectable. Another potential explanation is that although glucose-induced insulin transcription can start within minutes, the preproinsulin mRNA levels accumulate slowly against the high background of steady-state mRNA.

Many factors contribute to glucose-induced insulin transcription, including the transcription factors PDX-1 [79], MafA [39,80,81], and a heterodimer (E12/E47 and E2/5) and (BETA 2/NeuroD) [82,83] with the main glucose-responsive elements on the insulin promoter, the A3 [84], C1 and E1 [85] boxes. Insulin gene transcription is regulated through the activation of these factors and in combination, these factors can exert strong synergistic effects [37,86-88]. The upstream signaling that regulates these factors begins with β cell glucose metabolism.

The most important effects of glucose on insulin translation appear to be mediated by PDX-1. Glucose effects PDX-1 function in many different ways including a shift in the cellular distribution of PDX-1 from the cytoplasm to the nucleus, increases in the potential of the PDX-1 activation domain, and increases PDX1 binding to A3 [89-94]. The effects of glucose on PDX-1 are, in part, due to the phosphorylation of PDX-1 through the activation of phosphatidylinositol 3-kinase (PI3-K), while stress-activated protein kinase 2 (SAPK2/P38) may also be involved in this process [95], its function is controversial [96]. In fact, the PI3-K kinase pathway is considered a central regulator of PDX-1 and of glucose-induced insulin gene transcription [95,96]. Since PI3-K is a key molecule in the insulin receptor pathway, the activation of insulin gene transcription has been suggested, in part, to be the result of a feed forward mechanism involving the binding of secreted insulin to its receptor on the β cell surface [97-99].

Glucose and PDX-1 also modulate insulin transcription by influencing histones. When glucose levels are low, PDX-1 interacts with histone deacetylases Hdac-1 and Hdac-2, recruiting them to the insulin gene promoter where they deacetylate histone H4 and thereby down-regulate insulin gene expression [100].

Glucose also increases bHLH heterodimer binding to the E site. E47/BETA 2 is critical for glucose-induced insulin gene transcription [101-103]. Stimulatory glucose concentrations can activate β cell ERK1/2, and ERK1/2 promotes BETA 2 and E47 heterodimerization and binding to E-box sites [104]. Blocking ERK1/2 activity using kinase-defective ERK2 resulted in a substantial reduction in preproinsulin mRNA content after 24 hours[104]. Both the basal activity and the glucose-induced stimulation of the A2-E1 region of the insulin promoter are critically dependent upon ERK1/2 [105].

**Figure 4:**
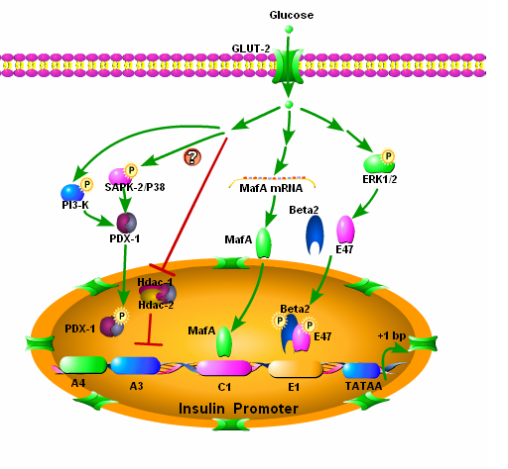
**Glucose control of insulin gene transcription**. Glucose metabolism in beta cells generates upstream signals that are responsible for the activation of factors involved in insulin transcription. (1) Glucose metabolism causes a shift of transcription factor PDX-1 from the cytoplasm to the nucleus, increases its activation domain and binding to A3 element. The effects are in part, due to the activity of phosphatidylinositol 3-kinase (PI3-K); another kinase stress-activated protein kinase 2 (SAPK2/P38) might be involved in this process. An alternative pathway involves histone and PDX-1. When glucose levels are low, PDX-1 interacts with histone deacetylases Hdac-1 and Hdac-2 and recruits them to insulin gene promoter, which causes the deacetylation of histone H4 and results in down-regulation of insulin gene expression. High concentrations of glucose diminish this inhibiting activity. (2) Stimulatory concentrations of glucose can activate ERK1/2, which promotes BETA2 and E47 heterodimerization and binding to E-box sites. (3) Glucose affects *MafA *at the mRNA level. Stimulatory glucose levels increase MafA transcription and result in increased MafA protein.

The mechanism by which MafA is activated by glucose is distinct. While glucose induces post translation modifications of PDX-1 and the bHLH heterodimer, glucose increases *MafA *transcription. At a glucose concentration of 2.8 mM, MafA expression is undetectable in whole β cell lysates. In contrast, at concentration of 16.7 mM MafA protein is readily detected. Exposing islets to 16.7 mM glucose for 24 hours also significantly increases *MafA *mRNA expression [89]. (Figure 4).

Immediate-early response genes (IEGs) are also involved with glucose mediated insulin biosythesis. IEGs are transcription factors that activate expression of downstream target genes, thus generating distinct biological responses by inducing specific long-term gene expression programs. In INS-1 β-like cells, glucose induces a Ca^2+^-dependent transcriptional activation of several immediate early genes such as *c-fos, c-jun, JunB, zif-268 *and *nur-77 *genes. In particular, c-fosand JunB proteins might facilitate glucose/cAMP mediated insulin gene induction [106]. 15 minutes of glucose stimulation induced *egr-1 *mRNA and protein synthesis, with maximum levels achieved in 30 minutes, in both glucose-responsive cell lines as well as primary islets. Glucose stimulation also induced expression of *c-fos *mRNA and *JunB *mRNA [107]. Thus, glucose metabolism could be coupled to transcriptional activation of immediate-early response factors.

Prolonged glucose stimulation leads to an increase in insulin transcription, which is supplementary to the glucose-induced translational control of proinsulin biosynthesis [77,108]. Under conditions of sustained secretory drive, stimulation of proinsulin gene transcription by glucose appears to be necessary for maintaining preproinsulin biosynthesis and hence conserving pancreatic insulin stores [108].

### Glucose control of translation

After insulin is secreted in response to glucose stimulation, renewed insulin biosynthesis begins immediately to replenish the insulin stores. After short term (less than 2 hours) glucose stimulation, insulin biosynthesis is mainly regulated at the translational level [55,109]. When primary rat islets are stimulated by high glucose for 1 hour, preproinsulin biosynthesis increases 4-5 fold without any change in the total preproinsulin mRNA level [77]. In another study, preproinsulin biosynthesis increased 25-fold when glucose increased from 1 to 10 mM [110].

The mechanism by which glucose regulates proinsulin synthesis in β-cells remains unclear. It was previously hypothesized that the insulin protein production increase resulted from increased translation initiation, elongation and signal recognition particle (SRP) release. Evidence has shown however that translation elongation occurs only at non-physiological glucose concentrations [56,111,112] and does not play a major role in glucose-stimulated insulin synthesis. Translation initiation is now thought to be the primary mechanism underlying glucose stimulated insulin synthesis.

Factors involved with glucose regulation of preproinsulin translation initiation are the translation initiation factors eIF4F, eIF2 and PERK [113,114]. The initiation complex eIF4F initiates the recruitment of the 40 S ribosome prerequisite to mRNA in protein synthesis. Hypophosphorylated eIF4E-binding proteins (4E-BPs) inhibit eIF4F complex formation but phosphorylation of 4E-BP1 decreases this inhibition, thereby increasing mRNA translation [46,47]. In primary islets and pancreatic β-cell lines, glucose increases 4E-BP1 phosphorylation [113], suggesting the eIF4F assembly importance in glucose-stimulated protein synthesis.

**Figure 5:**
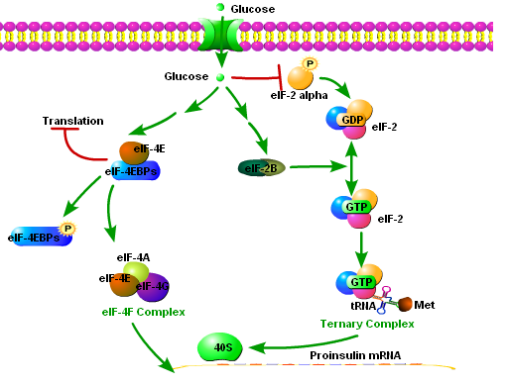
**Glucose control of insulin translation**. Glucose stimulates insulin synthesis largely by promoting insulin translation initiation. (1) Glucose promotes the phosphorylation of eIF-4EBP and activates eIF-4E; eIF-4E, eIF-4A and eIF-4G form eIF-4F, a complex whose functions include recognition of preproinsulin mRNA and recruiting 40S ribosome to mRNA. (2) eIF2 is a critical factor regulating protein biosynthesis. It is active only in GTP-bound state. A factor named eIF-2B functions to convert GDP-bound eIF-2 to GTP-bound eIF-2. The activity of eIF-2B is transiently up-regulated after glucose stimulation. Additionally, phosphorylation of alpha subunit of eIF2 (eIF2α) inhibits the formation of eIF2-GTP·Met-tRNAi translational ternary complex, which binds 40S ribosome and is indispensable for protein translation. Glucose causes the dephosphorylation of eIF2α, and induces an increase in the availability of the translational ternary complex.

Eukaryotic initiation factor 2 (eIF2) is another important factor regulating protein biosynthesis, participating in translational ternary complex (eIF2-GTP·Met-tRNAi) formation. This complex assembles only if eIF2 is in its GTP-bound state. After the formation of the 80S ribosome, the GTP bound to eIF2 is then hydrolyzed to GDP. The recycling of the inactive eIF2·GDP complex to the active eIF2·GTP complex is catalyzed by the guanine nucleotide exchange factor, eIF2B. eIF2B activity is transiently up-regulated in glucose stimulated isolated islets [111], suggesting that eIF2B may be an important modulator of insulin synthesis. Other studies suggest a role for the eIF2 alpha subunit (eIF2_α_); phosphorylation of eIF2_α _inhibits ternary complex formation, and glucose dephosphorylateseIF2_α _thereby increasing translational ternary complex availability [114] (Figure 5).

### Other genes and proteins affected by glucose

9Glucose also induces delayed long-term responses by inducing the expression of other genes involved in β-cell function such as glucose transporter 2 (GLUT-2), pyruvate kinase, acetyl-coenzyme A-carboxylase [115-118]. The expression of these genes is necessary to meet the increased metabolic and secretory demands during extended or repeated periods of hyperglycemia.

## Conclusion

Islet transplantation has the potential to benefit patients with type I diabetes, but it is a therapy limited by islet supply and other factors. While preliminary studies have found that stem cells can be induced to acquire a β-cell-like phenotype,, cells used for transplant therapy will need precisely replicate β-cell function. Characterizing the β-cell's unique molecular mechanisms underlying its glucose responsivity will allow investigators to better understand the critical elements stem cells must acquire and may allow investigators to better direct stem cell differentiation.

## Abbreviations

bHLH: basic helix-loop-helix proteins

eIF2: Eukaryotic initiation factor 2

Glut 2: glucose transporter 2

IEGs: Immediate-early response genes

K_ATP_-channels: ATP-sensitive potassium channels

L-VDCC: L-type voltage-dependent Ca^2+^channel

PDX-1: pancreas-duodenum homeobox-1

PERK: PKR-like ER kinase

PI-3-K: phosphatidylinositol (PI) 3-kinase

RRP: readily releasable pool

UTR: untranslated regions

4E-BPs: eIF4E-binding proteins
